# Cognitive impairment and associated risk factors in older adult hemodialysis patients: a cross-sectional survey

**DOI:** 10.1038/s41598-020-69482-1

**Published:** 2020-07-27

**Authors:** Yang Luo, Anne M. Murray, Yi-Dan Guo, Ru Tian, Peng-Peng Ye, Xin Li, Guo-Gang Li, Fang-Ping Lu, Ying-Chun Ma, Yi Sun, Yu-Zhu Wang, Yue-Fei Xiao, Qi-Meng Zhang, Xue-Feng Zhao, Hai-Dan Zhao, Xiang-Mei Chen

**Affiliations:** 1 Division of Nephrology, Beijing Shijitan Hospital, Capital Medical University, Bejing, 100038 China; 2 Berman Center for Clinical Research, Hennepin Health Research Institute, Hennepin Healthcare, and University of Minnesota, Minneapolis, MN 55455 USA; 3 Division of Injury Prevention and Mental Health National Center for Chronic and Non-Communicable Disease Control and Prevention, Chinese Center for Disease Control and Prevention, Room 916, No.27 Nanwei Road, Xicheng District, Beijing, 100053 China; 4 Department of Nephrology, Beijing Chaoyang Hospital, Capital Medical University, Beijing, 100032 China; 5 Department of Nephrology, Beijing Shijingshan Hospital, Beijing, 100047 China; 6 Department of Nephrology, Beijing Huaxin Hospital, the First Hospital of Tsinghua University, Beijing, 100084 China; 7 Department of Nephrology, China Rehabilitation Research Center Beijing Boai Hospital, Beijing, 100029 China; 8 Department of Nephrology, Beijing Fuxing Hospital Beijing, Capital Medical University, Beijing, 100036 China; 9 Department of Nephrology, Beijing Haidian Hospital, Beijing, 10058 China; 10 Department of Nephrology, Aerospace Central Hospital, Beijing, 100042 China; 11 Department of Nephrology, Beijing Zhongguancun Hospital, Beijing, 100586 China; 12 Department of Nephrology, Nankou Hospital of Beijing, Changping District, BeijingBeijing, 100024 China; 13 Department of Nephrology, Peking University Shougang Hospital, Beijing, 100574 China; 14 Department of Nephrology, Chinese PLA General Hospital, State Key Laboratory of Kidney Disease, Beijing, 100853 China

**Keywords:** Quality of life, Neurology

## Abstract

The clinical epidemiological features of cognitive impairment in Chinese older adult patients undergoing hemodialysis are not clear, we aimed to identify the extent and patterns of cognitive impairment among those patients. We conducted a cross-sectional study of 613 hemodialysis patients aged 50 to 80 from 11 centers in Beijing. A neuropsychological battery of 11 tests covering domains of attention/processing speed, executive function, memory, language, and visuospatial function was applied, patients were classified as none, mild, or major cognitive impairment according to the fifth version of the Diagnostic and Statistical Manual of Mental Disorders criteria for cognitive impairment. Compared with Chinese population norms, 37.2% of the participants had mild cognitive impairment, 43.7% had major cognitive impairment. Memory and language were the most severe impaired domains in the mild cognitive impairment group, attention and visuospatial function domains were the most serious impaired domains in the major cognitive impairment group. Concomitant impairment across multiple cognitive domains was common. Factors associated with major cognitive impairment included age, education level, history of stroke and hypertension, dialysis vintage, and single-pool Kt/V. There is a high frequency of cognitive impairment in Chinese older adult hemodialysis patients, with varying severity and concomitant impairment across multiple domains.

Cognitive impairment has become an important public health problem as the population ages, diabetes and cardiovascular disease prevalence continue to increase^1,2^. Reports from the USA and Italy show that cognitive impairment prevalence in hemodialysis patients ranged from 70 to 80% in the last 10 years, which were relatively higher than those individuals with normal kidney function^3,4^. Besides, previous studies have linked cognitive impairment with adverse outcomes that can potentially influence hemodialysis patient’s compliance regarding their dialysis schedules and medication regimens^5,6^.

According to the Chinese Renal Data System (https://www.cnrds.net), 447,435 patients with end-stage renal disease were undergoing hemodialysis by the end of 2016^7^. Previous studies in Chinese peritoneal dialysis patients showed that cognitive impairment, as defined by the Mini-Mental State Examination (MMSE), was present in 23 to 28%^8,9^. Since the MMSE does not include sensitive measures of attention and executive function, these studies may not have adequately demonstrated more subtle features of cognitive impairment in dialysis patients. Currently, no studies have systematically measured cognitive function across multiple domains in patients undergoing hemodialysis in China.

To fill this gap, we conducted a cross-sectional analysis to assess the features of cognitive impairment using a neuropsychological battery that covered five cognitive domains (attention/processing speed, executive function, memory, language, and visuospatial function) in 613 older adult hemodialysis patients and compared the data with age-matched general Chinese population norms. We also identified factors associated with cognitive impairment in these patients.

## Results

### Demographics and clinical variables of the patients

Of the 670 included HD patients, 57 were eligible but excluded for lacking patient interest for the comprehensive cognitive tests, these 57 excluded patients had similar demographic and clinical characteristics (*p* > 0.05) (Supplemental Table S1). In the remaining 613 patients provided consent to participate in the full cognitive test battery, the mean age of the participants was 63.82 ± 7.14 years, 42.1% were women, 91.4% were married, and only 5.9% had less than six years of education. Hypertension (88.9%), diabetes (37.7%), and coronary heart disease (31.5%) were the most common diseases in medical history. The dialyzers with polyacrylonitrile, polysulfone, polycarbonate membranes used in the hemodialysis treatment were 65.5%, 23.4%, and 11.1%, respectively. The average treatment session length was 3.81 ± 0.27 h. Patients with cognitive impairment were more likely to be older; have a lower education level; a longer hemodialysis vintage; comorbidities of diabetes, hypertension, and stroke; a higher level of serum iPTH; and lower level of single-pool Kt/V. lower MoCA-BJ scores and higher ADL scores (Table 1).

**Table 1:** Demographic and clinical characteristics of the study participants. Data were presented as mean ± SD or median (interquartile range) for continuous variables, and number (%) for categorical variables. *Abbreviation*: CHD, coronary heart disease; HF, heart failure; BMI, body mass index; Kt/V, an indicator for evaluating dialysis adequacy; Hb, hemoglobin; ALB, albumin; CRP, C-reactive protein; iPTH, intact parathyroid hormone; MoCA-BJ, the Chinese Beijing version of the Montreal Cognitive Assessment; ADL, activities of daily living.

Characteristics	Total (n = 613)	Cognitive impairment			*p*
		None (n = 117)	Mild (n = 228)	Major (n = 268)	
Age, years	63.82 ± 7.14	59.29 ± 7.71	63.82 ± 7.14	65.41 ± 7.62	< 0.001
Gender, female	258 (42.1%)	47 (40.2%)	93 (40.8%)	118 (44.0%)	0.695
**Marital status**					0.773
Single	53 (8.6%)	10 (8.5%)	22 (9.6%)	21 (7.8%)	
Married	560 (91.4%)	107 (91.5%)	206 (90.4%)	247 (92.2%)	
**Education level**					0.002
< 6 years	36 (5.9%)	6 (5.1%)	11 (4.8%)	19 (7.1%)	
6–12 years	408 (66.5%)	63 (53.8%)	169 (74.1%)	175 (65.2%)	
> 12 years	169 (27.6%)	48 (41.0%)	48 (21.1%)	74 (27.7%)	
**Smoking history**					0.783
Never	343 (56.0%)	66 (56.4%)	121 (53.1%)	156 (58.2%)	
Former	186 (30.3%)	34 (29.1%)	76 (33.3%)	76 (28.4%)	
Current	84 (13.7%)	17 (14.5%)	31 (13.6%)	36 (13.4%)	
**Alcohol intake**					0.226
Never	352 (57.4%)	66 (56.4%)	123 (53.9%)	163 (60.8%)	
Former	233 (38.0%)	45 (38.5%)	90 (39.5%)	98 (36.6%)	
Current	28 (4.6%)	6 (5.1%)	15 (6.6%)	7 (2.6%)	
**Medical history**					
Diabetes	231 (37.7%)	33 (28.2%)	84 (36.8%)	114 (42.5%)	0.027
Hypertension	545 (88.9%)	95 (81.2%)	203 (89.1%)	250 (93.3%)	0.004
Stroke	100 (16.3%)	6 (5.1%)	38 (16.7%)	56 (20.9%)	0.001
CHD	193 (31.5%)	36 (30.8%)	68 (29.8%)	89 (33.2%)	0.709
HF	104 (17.0%)	20 (17.1%)	40 (17.5%)	44 (16.4%)	0.975
BMI, kg/m^2^	23.60 ± 4.11	24.21 ± 5.78	23.29 ± 3.53	23.60 ± 3.69	0.171
Dialysis vintage, mo	57.00 (24.00, 101.50)	43.00 (12.00, 87.75)	57.00 (20.75, 108.00)	65.00 (32.00, 103.00)	< 0.001
Single-pool Kt/V	1.29 ± 0.18	1.37 ± 0.15	1.30 ± 0.19	1.24 ± 0.16	< 0.001
Hb, g/L	11.11 ± 1.46	11.07 ± 1.53	11.11 ± 1.64	11.13 ± 1.27	0.938
Alb, g/L	39.93 ± 3.18	40.25 ± 2.55	39.99 ± 3.20	39.75 ± 3.38	0.429
CRP, mg/L	2.60 (1.19, 7.05)	2.37 (1.21, 6.04)	2.79 (1.19, 6.35)	2.70 (1.15, 7.58)	0.807
Calcium, mmol/L	2.24 ± 0.25	2.21 ± 0.22	2.28 ± 0.25	2.22 ± 0.25	0.018
Phosphate, mmol/L	1.72 ± 0.65	1.77 ± 0.68	1.67 ± 0.61	1.73 ± 0.67	0.337
iPTH, pg/mL	271.17 ± 249.85	224.32 ± 200.92	245.97 ± 199.49	313.06 ± 297.41	0.001
Depression score	4.83 ± 5.08	5.17 ± 5.45	5.10 ± 5.40	4.44 ± 4.57	0.300
MoCA-BJ score	21.78 ± 3.85	25.74 ± 2.39	23.59 ± 1.40	18.52 ± 3.10	< 0.001
ADL total score	20.38 ± 7.30	15.50 ± 1.31	16.54 ± 2.14	25.78 ± 8.07	< 0.001
Basic ADL score	7.53 ± 1.81	6.56 ± 0.55	6.90 ± 1.04	8.48 ± 2.19	< 0.001
Instrument ADL score	12.85 ± 5.75	8.95 ± 0.85	9.64 ± 1.61	19.29 ± 6.17	< 0.001

### Performance on cognitive function tests

The raw scores and percentages of the 613 hemodialysis patients who scored less than 1.50 SDs, between 1.50 and 1.99 SDs, and 2.00 or more SDs below the age- and education-adjusted population norm for each test are presented in Table 2. Among the 11 cognitive tests, 32.79% of patients scored 2.0 or more SDs below the population norm on the CFT-copy test for the visuospatial function domain, and 29.04% and 24.80% of patients scored 2.0 or more SDs below the population norm on the SDMT and TMT-A tests for the attention/processing speed domain, respectively. For the six tests measuring the memory and executive function domains, 9 to 19% scored 2.0 or more SDs below the population norms, only less than 2% on the AFT and BNT for the language domain score.

**Table 2:** Cognitive tests raw scores and the percentage by the number of SDs below adjusted population norms. Data were shown as mean ± SD or frequencies (%). *Abbreviation:* AVLT, Auditory Verbal Learning Test; CFT, Rey-Osterrieth Complex Figure; SDMT, Symbol Digit Modalities Test; TMT-A, Trail Making Test A; TMT-B, Trail Making Test B; SCWT, Stroop Color-Word Test; AFT, Animal Fluency Test; BNT, Boston Naming Test.

Domains	Tests	Raw-scores (n = 613)	Percentage by number of SDs below adjusted population norms (%)		
			< 1.50 SD (n = 117)	1.50–1.99 SD (n = 228)	≥ 2.0 SD (n = 268)
Attention/processing speed	SDMT	26.69 ± 12.32	42.58	28.38	29.04
	TMT-A	87.17 ± 53.11	58.89	16.31	24.80
Executive function	TMT-B	174.80 ± 74.73	71.29	9.79	18.92
	SCWT-C	44.49 ± 7.51	86.62	4.24	9.14
	SCWT-T	105.07 ± 50.42	67.70	14.68	17.62
Memory	AVLT 5	4.92 ± 2.90	74.71	14.03	11.26
	AVLT1-5	28.23 ± 11.93	78.79	11.58	9.62
	CFT- memory	13.41 ± 8.32	71.78	13.70	14.52
Language	BNT	27.54 ± 3.04	93.64	4.89	1.47
	AFT	17.13 ± 5.33	95.43	2.94	1.63
Visuospatial function	CFT- copy	27.78 ± 8.88	57.75	9.46	32.79

### Frequency of cognitive impairment

Of the 613 hemodialysis patients, global cognitive impairment was detected in 496 (80.91%) (95% CI 77.80%, 84.02%); with 228 (37.19%) (95% CI 33.36%, 41.02%) classified with mild cognitive impairment, 268 (43.72%) (95% CI 39.79%, 47.65%) with major cognitive impairment, and only 117 (19.09%) (95% CI 15.98%, 22.20%) with normal cognition, according to the fifth version of the Diagnostic and Statistical Manual of Mental Disorders (DSM-V) criteria.

### Comparison of impairment across five cognitive domains with standard T-scores

The standardized T-scores for each of the five cognitive domains are shown in Table 3. One-way ANOVA results revealed significant differences in performance within each domain across the three levels of cognitive impairment. Least significant difference (LSD) adjustment for multiple comparisons revealed that cognitive function, expressed by T-scores, decreased significantly in all domains (*p* < 0.001), except for the visuospatial function domain between the no cognitive impairment and mild cognitive impairment groups (*p* = 0.288). The memory and language domains were the most seriously impaired domains compared with the other domains in the mild cognitive impairment group (*p* < 0.001), and the visuospatial function and attention/processing speed domains were the most seriously impaired domains compared with other domains in the major cognitive impairment group (*p* < 0.001) (Table 3).

**Table 3:** Performance using T scores by cognitive domains for each level of cognitive impairment. Data were expressed as mean ± SD. T-score was a standard score Z shifted and scaled to have a mean of 50 and a standard deviation of 10. Higher scores are consistent with better performance on all cognitive domains.

Domains	All (n = 613)	Cognitive impairment			*p*^a^
		Non (n = 117)	Mild (n = 228)	Major (n = 268)	
Memory	50.00 ± 6.46	55.21 ± 5.12^b^	51.63 ± 5.24^b^	46.33 ± 5.77^b^	< 0.001
Attention/processing speed	50.00 ± 8.82	57.64 ± 4.84^b^	53.46 ± 5.21^b^	43.72 ± 8.33^b^	< 0.001
Executive function	50.00 ± 7.21	55.71 ± 3.47^b^	53.10 ± 3.89^b^	44.87 ± 7.24^b^	< 0.001
Language	50.00 ± 8.13	54.29 ± 5.83^b^	52.53 ± 6.27^b^	45.98 ± 8.57^b^	< 0.001
Visuospatial function	50.00 ± 10.00	56.33 ± 2.19^c^	55.41 ± 2.83^c^	42.64 ± 11.11^b^	< 0.001

^a^*p* value for one-way ANOVA F test, the null hypothesis is that mean values are equal for all 3 cognitive groups.

^b^Designate significant difference from any other cognitive group values in the same row (after least significant difference (LSD) adjustment for multiple comparisons).

^c^Designate nonsignificant differences in the visuospatial function domain between the non- and mild cognitive impairment groups (*p* = 0.288).

### Patterns of impairment co-occurrence across multiple cognitive domains

**Figure 1:**
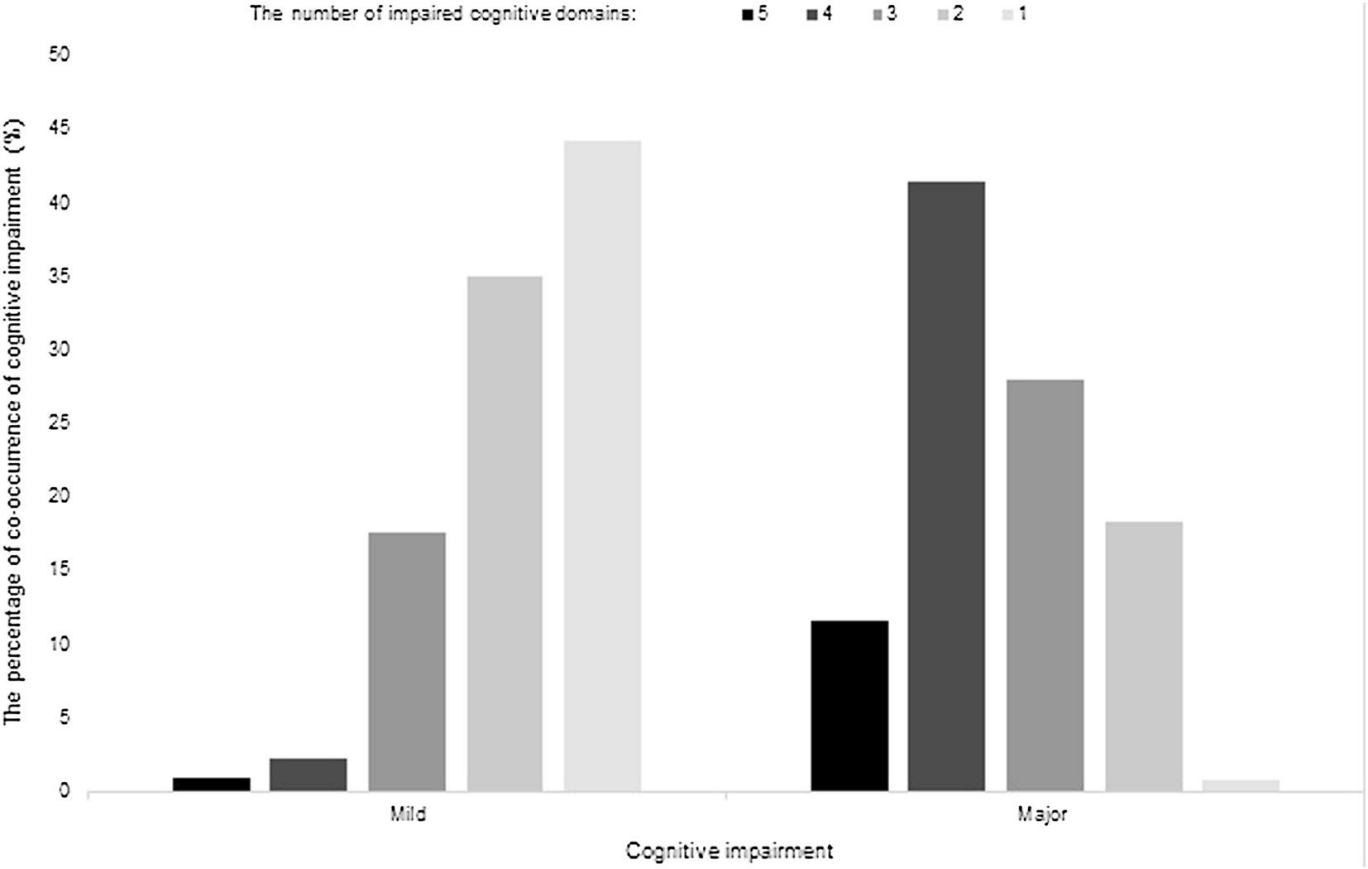
Distribution of co-occurrence impairment across multiple domains in mild and major cognitive impairment groups.

The co-occurrence of impaired cognitive function across domains was common among study participants. Only 117 patients (19.09%) had no impairment in any domain. The most common pattern in the mild cognitive impairment group was single-domain impairment (44.30%); the proportions of two-, three-, four-, and five-domain impairments were 35.09%, 17.54%, 2.19%, and 0.88%, respectively. In the major cognitive impairment group, the proportions of two-, three-, four-, and five-domain co-occurrence impairments were 18.28%, 27.99%, 41.42%, and 11.57%, respectively (*p* < 0.001), while the proportion of a single cognitive domain impairment was only 0.75%, lower than that of the mild cognitive impairment group (*p* < 0.001) (Fig. 1).

### Factors associated with cognitive impairment

In the fully adjusted logistic regression model, factors associated with mild cognitive impairment included age, comorbidity of stroke, hemodialysis vintage, and single-pool Kt/V. Factors associated with major cognitive impairment included age, 12 or more years of education, comorbidities of stroke and hypertension, hemodialysis vintage, and single-pool Kt/V (Table 4).

**Table 4:** Risk factors associated with cognitive impairment. *Models were adjusted for age, sex, education level, smoking history, alcohol intake, comorbidities, hemodialysis vintage, Kt/V and the serum level of Hb, ALB, CRP, and iPTH. *Abbreviation*: OR, odds ratio; CI, confidence interval; HD, hemodialysis; Kt/V, an indicator for evaluating dialysis adequacy; Hb, hemoglobin; ALB, albumin; CRP, C-reactive protein; iPTH, intact parathyroid hormone.

Variables	Mild cognitive impairment (n = 228)		Major cognitive impairment (n = 268)	
	OR (95% CI)*	*p*	OR (95% CI)*	*p*
Age (per 1-y increase)	1.09 (1.05, 1.13)	< 0.001	1.12 (1.08, 1.16)	< 0.001
Male sex	0.92 (0.55, 1.53)	0.735	0.99 (0.59, 1.68)	0.981
**Education level**				
< 6 years	1.00 (Reference)		1.00 (Reference)	
6 ~ 12 years	1.28 (0.42, 3.94)	0.669	0.69 (0.23, 2.05)	0.502
> 12 years	0.38 (0.12, 1.21)	0.100	0.31 (0.10, 0.94)	0.039
**Smoking history**				
Never	1.00 (Reference)		1.00 (Reference)	
Former	0.87 (0.43, 1.77)	0.699	0.53 (0.25, 1.10)	0.091
Current	0.56 (0.24, 1.30)	0.178	0.48 (0.20, 1.13)	0.089
**Alcohol intake**				
Never	1.00 (Reference)		1.00 (Reference)	
Former	1.34 (0.42, 4.30)	0.618	0.86 (0.44, 1.67)	0.653
Current	0.91 (0.48, 1.75)	0.786	0.44 (0.12, 1.68)	0.232
Hypertension	1.40 (0.68, 2.88)	0.367	2.70 (1.22, 5.99)	0.015
Diabetes	1.60 (0.90, 2.86)	0.112	1.73 (0.96, 3.10)	0.069
Stroke	3.35 (1.28, 8.76)	0.014	4.27 (1.63, 11.19)	0.003
HD vintage (per 1-mon increase)	1.02 (1.01, 1.02)	< 0.001	1.02 (1.01, 1.02)	< 0.001
Single-pool Kt/V (per 1 increase)	0.04 (0.01, 0.19)	< 0.001	0.003 (0.000, 0.013)	< 0.001
Hb (per 1-g/L increase)	0.99 (0.98, 1.02)	0.947	0.99 (0.97, 1.02)	0.645
ALB (per 1-g/L increase)	0.99 (0.89, 1.07)	0.897	0.92 (0.83, 1.03)	0.132
CRP (per 1-mg/L increase)	1.01 (0.98, 1.05)	0.548	0.97 (0.92, 1.01)	0.117
iPTH (per 1-pg/mL increase)	1.00 (0.99, 1.02)	0.934	1.00 (0.99, 1.03)	0.194

### Sensitivity analyses

Stratifying patients according to hemodialysis vintage (< 36 months and ≥ 36 months) or single-pool Kt/V (< 1.2 and ≥ 1.2) did not significantly change the influence of the age and hemodialysis vintage factors, which were still significantly associated with higher risks of either mild or major cognitive impairment in the multivariate-adjusted analyses. However, the associations between cognitive impairment and education level, history of hypertension or stroke, and single-pool Kt/V were affected to varying degrees, and in some cases the association became non-significant. With dialysis vintage beyond 36 months, the diabetes comorbidity became a risk factor for cognitive impairment (Supplemental Tables S2 and S3). After excluding 45 individuals with depression scores of 7 or higher, results were essentially unchanged in the remaining 568 participants.

## Discussion

In this cross-sectional study of 613 older adult hemodialysis patients, we found that the frequency of cognitive impairment in Chinese hemodialysis patients was 80.9%, very similar to the 87.3% frequency estimate in a similar study of 374 hemodialysis patients in the US age 55 and older^10^. This high frequency of cognitive impairment is nearly four times as much as that in the Chinese community-based studies of individuals age 60 and older^11^. Impairment severity tended to differ across domains in the mild and major cognitive impairment groups, and co-occurrence of impairments across multiple cognitive domains occurred in 57.8% and 99.3% of patients in the mild and major cognitive impairment groups, respectively. Apart from previously known associated factors, such as age, education level, and history of stroke and hypertension, we also demonstrated that some hemodialysis-related factors, such as dialysis vintage and single-pool Kt/V, were correlated with cognitive impairment.

Our study was one of the largest to date to focus on the issue of cognitive impairment in older adult hemodialysis patients. In a previous cross-sectional study, Murray measured cognitive function in 374 hemodialysis patients age 55 and older with an age-matched general population group and reported an 87.3% occurrence of cognitive impairment associated with low education, higher Kt/V, and history of stroke^10^. In another study evaluating cognitive function in 676 hemodialysis patients in Italy, cognitive impairment occurred in 70.1% of participants, and the co-occurrence patterns of impairment across domains were similar to findings in the present study, with only 25.9% of participants impaired on a single cognitive domain^4^. The results of our study were also consistent with previous studies that demonstrated a high prevalence of cognitive impairment in hemodialysis patients, it also indicates that cognitive impairment has become a critical public health issue among Chinese hemodialysis patients.

Delineating the features of cognitive impairment in Chinese hemodialysis patients was another objective of our study. Before the DSM-V standard was published, some studies investigated the impaired domains of cognitive function in hemodialysis patients and found that the executive and memory domains were the most impaired domains^12^. Sarnak^13^ evaluated the cognitive function of 314 hemodialysis patients from six Boston-area hemodialysis units and found that the patients on dialysis had a significantly poorer executive function, but not memory performance, compared to population norms. This result supported the hypothesis that vascular disease, whether due to atherosclerosis or arteriosclerosis, may be the primary cause of cognitive impairment in hemodialysis patients. A recently published meta-analysis of 42 studies covering 3,522 participants found that people treated with hemodialysis had worse cognition, particularly in attention/processing speed and memory than the general population^14^. In our assessment, the impairments in memory and language domains tended to be more serious than the other three domains in the mild cognitive impairment group, this might be the reason why the mild cognitive impairment was not easy to be diagnosed in the early stage. We also found that the attention/processing speed and visuospatial domains were the most seriously impaired domains in the major cognitive impairment group, which could be an explanation about the negative influence of the quality of life in those patients. These results suggested that there were different impairment features in mild and major cognitive impairment groups of hemodialysis patients. Compared with above-mentioned studies, the cognitive impairment in our patients mainly focused on the memory and language attention/processing speed, and visuospatial function domains. These detailed features of cognitive impairment in Chinese hemodialysis patients also provide basic information for future studies to explore the mechanism of cognitive impairment in these patients.

Previous studies indicated that hemodialysis patients are at increased risk of cognitive impairment because of their older age, low level of education, and a high prevalence of cardiovascular risk factors^3,5,15^. However, the risk factors associated with cognitive impairment are not clear, especially concerning the role of some hemodialysis-related factors^3^. Our results showed that older age, comorbidities of stroke, longer hemodialysis vintage, and decreased single-pool Kt/V were independent risk factors for both mild and major cognitive impairment, beside these, lower education and hypertension were independent risk factors for major cognitive impairment, the reason of the different risk factors between these two groups was still not clear , although these factors were heterogeneous and overlap in mild and major cognitive impairment groups, they were potentially belongs to the neurodegenerative component and need to be explored in the future study. We have noticed that these risk factors, validated by our study, were consistent with previous studies except for one important index, single-pool Kt/V, which could reflect the clearance of small molecular weight toxins in hemodialysis. In contrast to our results, the previous study showed that higher Kt/V was associated with worse rather than better cognitive function^3^. Potential explanations for this counterintuitive association include selection bias, in which patients with greater cognitive impairment are dialyzed more intensively, or that repeated rapid removal of solutes might have previously unappreciated cumulative detrimental effects on cognition. However, the DOPPS study, which was designed to improve health and longevity of patients with ESRD, has generally supported the observation that a Kt/V less than 1.2 is associated with increased mortality among hemodialysis patients, and lower Kt/V is also associated with anemia and malnutrition among those patients^16,17^. We also conducted a sensitivity analysis by stratifying patients by hemodialysis vintage (< 36 months and ≥ 36 months) or single-pool Kt/V (< 1.2 and ≥ 1.2), but the results still showed that lower Kt/V was associated with cognitive impairment. Our findings, coupled with previous findings, indicated that both higher and lower doses of hemodialysis might be correlated with cognitive impairment in hemodialysis patients.

Our study has several strengths. It includes a relatively large cohort and incorporated detailed ascertainment of both cognitive impairment and its potential risk factors in 613 patients selected from 11 hemodialysis centers in Beijing. Our study population appears to be representative of the general Chinese older adult hemodialysis population, as the characteristics of those patients described by the Chinese Renal Data System (https://www.cnrds.net). For example, the primary cause of renal failure (diabetes and hypertension), dialysis vintage, and serum hemoglobin levels, were not significantly different^18^. Thus our results are likely generalizable to the overall Chinese older hemodialysis population. Cognitive assessment in our study consisted of a standardized and validated battery covering five cognitive domains, with strong reliability and criterion validity linked with Chinese population norms. Besides, cognitive impairment classification was based on the latest published criteria from DSM-V. Patients were assessed by well-trained and certified research staff to protect the accuracy of the assessment data. To the best of our knowledge, this is the first study to offer a detailed assessment of cognitive function among Chinese hemodialysis patients. However, our study also had several important limitations. First, our study was designed as cross-sectional analysis, it is not possible to determine whether the associated factors with cognitive impairment were causal, and some unrecognized risk factor like medication were also not included in our study. Second, since we excluded short dialysis vintage patients and patients with sensory (visual and hearing) or motor impairment, the cognitive impairment frequencies in our study likely underestimate the true prevalence of cognitive impairment among hemodialysis patients. Third, we excluded patients who had sensory (e.g., visual and hearing) or motor impairment, or whose estimated life expectancy was less than six months, this might have led to an underestimation of cognitive dysfunction, the assessment of cognitive function in such special groups of the population has caused attention in recent years^19^, creating nonvisual and nonverbal cognitive tests would be a valuable solution in the future for those population.

In conclusion, our findings suggest a high frequency of cognitive impairment across a broad spectrum of cognitive domains in Chinese older adult hemodialysis patients. Impairment severity varied across different domains and co-occurrence of multiple-domain cognitive impairments is common and diverse. Apart from previously known associated factors, such as age, education status, and history of stroke and hypertension, dialysis vintage and dialysis dose were also correlated with cognitive impairment in these patients. These results have important implications for clinicians caring for dialysis patients given their increased likelihood of non-compliance with medications and dietary recommendations due to their high prevalence of cognitive impairment.

## Methods

### Study design and participants

This cross-sectional analysis used the data repository from the observational cohort study of cognitive impairment in Chinese patients undergoing hemodialysis (Registered in ClinicalTrials.gov, ID: NCT03251573). Potential study participants were chronic hemodialysis patients recruited from 11 hemodialysis centers in Beijing, who were screened for eligibility between April 2017 and June 2017. The eligibility criteria were as follows: (1) age 50 to 80 years, (2) diagnosed with end-stage kidney disease, (3) treated with long-term outpatient hemodialysis for a minimum of three months, (4) willing to provide written informed consent, (5) ability to complete a 90 min cognitive and physical function battery, and (6) the patient’s first language was Chinese. The exclusion criteria for all participants were: (1) unable to participate for reasons such as sensory (e.g., visual and hearing) or motor impairment, (2) estimation of a life expectancy of six months or less according to the nephrologists, and (3) recently diagnosed with major psychiatric disorders (e.g., psychosis, depression).

### Standard protocol approvals, registrations, and patient consents

This research was conducted under the ethical standards described by the Declaration of Helsinki and approved by the Institutional Ethical Review Board of Beijing Shijitan Hospital, Capital Medical University (approval no. SJT2016-18), this approved certificate in the principal investigator’s hospital is also authorized by other joining hospitals as a general ethical document. All participants who completed the detailed cognitive testing provided written informed consent unless there was major cognitive impairment, in which case the participant’s legal guardians signed the consent form.

### Neuropsychological assessment

All neuropsychological assessments were performed by the research staff that were centrally trained and certified by the study neuropsychologist to conduct the assessments before study commencement. All research staff and patients were native Chinese speakers. To avoid the influence of hemodynamic changes in the dialysis treatment day, a neuropsychological assessment was conducted individually in a separate room on the day after a dialysis session and required on average approximately 90 min. Global cognitive function was screened by the Chinese Beijing version of the Montreal Cognitive Assessment (MoCA-BJ)^20^. The comprehensive battery of neuropsychological tests was designed to assess five cognitive domains: (1) attention/processing speed, using Symbol Digit Modalities Test (SDMT) and the Chinese modified version of the Trail Making Test A (TMT-A)^21,22^; (2) executive function, using the Chinese modified version of the Trail Making Tests B (TMT-B), and a modified version of the Stroop Color-Word Test (SCWT)^23,24^; (3) verbal memory, using the Chinese version of the Auditory Verbal Learning Test (AVLT) for short-delay and long-delay free recall and Complex Figure for visual memory (delayed recall test; Chinese version)^25^; (4) language, using the Chinese modified versions of Boston Naming Test (BNT) and Animal Fluency Test (AFT)^26^; and (5) visuospatial function, using the Rey-Osterrieth Complex Figure (CFT)^27^. Depression was assessed using the Hamilton Depression Scale, with scores ranging from 0 to 63 and a score of 7 or above suggested as the optimal cutoff for suspected depression^28^. The functional status was evaluated using Lawton and Brody instrumental activities of daily living scales, including 6 basic items and 8 instrumental items. Four levels of activities of daily living (1 = independent; 2 = need supervision; 3 = need help; 4 = unable) were used for evaluation^29^.

### The cognitive impairment classification algorithm

We classified subjects as having no, mild, or major cognitive impairment using criteria from DSM-V as a guideline^30^. The age and/or education-adjusted raw score of each cognitive test was calculated and compared with the age and/or education-adjusted published norms for Chinese populations^31–34^. Specifically, age- and education-adjusted scores less than 1.5 standard deviations (SDs) below the adjusted mean of the published population norms on all tests in all domains indicated no cognitive impairment; scores of 1.50 to 1.99 SDs and 2.0 or more SDs below the adjusted mean of the published norms on at least one test in at least one domain indicated mild cognitive impairment and major cognitive impairment, respectively^30,35^.

### Factors associated with cognitive impairment

Demographic, clinical, and laboratory factors were ascertained at the time of cognitive testing. Sociodemographic information, medical history, health behaviors, and hemodialysis vintage were obtained by participant self-report and patients’ electronic or paper charts. Height, weight, and blood pressure of the enrolled HD patients were recorded by trained study personnel on the same day of receiving the baseline neurological tests. Pre-dialysis blood tests included measurement of the serum levels of hemoglobin, albumin, phosphate, intact parathyroid hormone (iPTH), and novel cardiovascular risk factors (i.e., C-reactive protein [CRP]); the single-pool Kt/V was calculated from the pre-and post-dialysis serum urea nitrogen levels.

### Statistical analysis

Data are presented as the mean ± SD for continuous variables with a normal distribution, medians and interquartile ranges for continuous variables without a normal distribution, and proportions for categorical variables. One-way analysis of variance (ANOVA), Kruskal–Wallis tests and Chi-square tests were used to compare the demographics, clinical characteristics, and five cognitive domain scores stratified by level of cognitive impairment.

We calculated the frequency of mild and major cognitive impairment based on the raw scores of each cognitive test and the percentage of scores that were less than 1.50 SDs, between 1.50 and 1.99 SDs, and 2.0 or greater SDs below the adjusted population norms.

To compare the difference of cognitive impairment of each domain among the three cognitive groups, the raw score of each neuropsychological test was transformed into a T-score (a standard Z score shifted and scaled to have a mean of 50 and a standard deviation of 10), and then the T-scores for each domain were generated by averaging the T-scores of their respective tests. Least significant difference (LSD) adjustment for multiple comparisons were conducted. We also compared impairment co-occurrence across multiple cognitive domains between the mild and major cognitive impairment groups.

We used multivariable logistic regression analysis to evaluate the factors associated with mild or major cognitive impairment versus no cognitive impairment. Variables associated with cognitive impairment on unadjusted analyses with *p* ≤ 0.10 and potential clinical risk factors for cognitive impairment were entered into the logistic regression model as covariates, with mild and major cognitive impairment as the dependent variables, adjusted for age, sex, education level, smoking and alcohol intake, comorbidities including the medical history of hypertension, diabetes, depression status, and stroke, hemodialysis vintage, Kt/V, and the serum levels of Hb, ALB, iPTH, and CRP. All analyses were conducted with Stata version 14.2 (STATA, College Station, TX), using two-tailed 95% confidence intervals (CI). *p* values < 0.05 were considered statistically significant.

### Sensitivity analyses

Previous studies have reported that dialysis vintage and Kt/V may be associated with cognitive function impairment, so we performed sensitivity analyses to validate the stability of our study findings depending on these two factors^10^. First, we stratified the participants into two dialysis vintage groups (< 36 months and ≥ 36 months). In the second sensitivity analysis, we stratified the participants into two single-pool Kt/V groups (< 1.2 and ≥ 1.2). Besides, since depression may affect cognitive function, the analysis was repeated excluding patients with a Hamilton Depression score of 7 or greater.

## Supplementary information

Supplementary file 1.

## Data Availability

All data related to this article are shown in the manuscript or are available upon request from the corresponding authors.
