# Supplementary material for: Cognitive impairment and associated risk factors in older adult hemodialysis patients: a cross-sectional survey
Source: Sci Rep. 2020 Jul 27;10:12542. doi: 10.1038/s41598-020-69482-1 (PMC7385128; doi:10.1038/s41598-020-69482-1)
Supplement: Supplementary file 1 [file 41598_2020_69482_MOESM1_ESM.pdf]

Cognitive impairment and associated risk factors in older adult hemodialysis patients: A cross-sectional survey

Yang Luo[1,*], Anne M Murray [2], Yi-Dan Guo[3], Ru Tian[4], Peng-Peng Ye [5], Xin Li[6], Guo-Gang Li[7], Fang-Ping Lu[8], Ying-Chun Ma[9], Yi Sun[10], Yu-Zhu Wang[11], Yue-Fei Xiao[12], Qi-Meng Zhang[13], Xue-Feng Zhao[14], Hai-Dan Zhao[15], Xiang-Mei Chen[16*]

*** Co-correspondence to:**

Yang Luo, MD,

Department of Nephrology, Beijing Shijitan Hospital, Capital Medical University.

10 Tieyi Road, Haidian District, Beijing, 100038, China.

Phone: +86-010-63926891

Fax: +86-010-63926891

Email: luoyang96@163.com

Xiangmei Chen, MD

Department of Nephrology, Chinese PLA General Hospital, State Key Laboratory of Kidney Disease, 28 Fuxing Road, Beijing 100853, China.

Phone: +86-010-66935462

Fax: +86-010-68130297

E-mail: xmchen301@126.com

Characteristics	**Included** (n=613)	**Excluded** (n=57)	***P***
Age (years)	63.82±7.14	64.61±8.50	0.4
Female sex	258(42.1%)	24(42.1%)	0.9
Marital status			0.9
Single	53(8.6%)	6(10.5%)	
Married	560(91.4%)	51(89.5%)	
Education level			0.01
<6 years	36(5.9%)	25(43.9%)	
6-12 years	408(66.5%)	32(56.1%)	
>12 years	169(27.6%)	0(0%)	
Smoking history			0.7
Never	343(56.0%)	32(56.2%)	
Former	186(30.3%)	15(26.3%)	
Current	84(13.7%)	10(17.5%)	
Alcohol intake			0.04
Never	352(57.4%)	32(56.1%)	
Former	233(38.0%)	18(31.6%)	
Current	28(4.6%)	7(12.3%)	
Comorbidities			
Diabetes	231(37.7%)	15(26.3%)	0.09
Hypertension	545(88.9%)	53(93.0%	0.3
Stroke	100(16.3%)	5(8.8%)	0.1
CHD	193(31.5%)	18(31.6%)	0.9
HF	104(17.0%)	4(7.0%)	0.7
BMI (kg/m^2)	23.60±4.11	24.09±3.73	0.4
Dialysis vintage (mo.)	57.00(24.00, 101.50)	63.50(21.25, 100.50)	0.5
Single-pool Kt/V	1.29±0.18	1.27±0.20	0.8
Hb (g/L)	11.11±1.46	11.02±1.49	0.6
Alb (g/L)	39.93±3.18	40.29±2.61	0.4
CRP (mg/L)	2.60(1.19, 7.05)	1.77(0.99, 4.67)	0.1
Phosphate (mmol/L)	1.72±0.65	1.71±0.68	0.9
iPTH (pg/mL)	271.17±249.85	244.00±172.97	0.3

Table S1. Demographics and clinical Characteristics of included and excluded patients. Data were presented as mean ± SD or median (interquartile range) for continuous variables, and number (%) for categorical variables. *Abbreviation*: CHD, coronary heart disease; HF, heart failure; BMI, body mass index; Kt/V, an indicator for evaluating dialysis adequacy; Hb, hemoglobin; ALB, albumin; CRP, C-reactive protein; iPTH, intact parathyroid hormone.

Variables	Hemodialysis vintage <36 months (n=204)				Hemodialysis vintage ≥ 36 months (n=409)			
	Mild cognitive impairment (n=69)		Major cognitive impairment (n=78)		Mild cognitive impairment (n=159)		Major cognitive impairment (n=190)	
	OR (95% CI)*	*P*	OR (95% CI)*	*P*	OR (95% CI)*	*P*	OR (95% CI)*	*P*
Age (per 1-y increase)	1.11(1.05, 1.19)	0.001	1.15(1.08, 1.23)	<0.001	1.11(1.05, 1.17)	0.01	1.14(1.08, 1.21)	<0.001
Male sex	1.55(0.64, 3.74)	0.3	0.89(0.35, 2.23)	0.8	0.75(0.38, 1.48)	0.4	1.02(0.52, 2.03)	0.9
Education level								
<6 y	1.00(Reference)		1.00(Reference)		1.00(Reference)		1.00(Reference)	
6~12 y	2.58(0.22, 29.65)	0.4	0.28(0.04, 1.79)	0.2	1.21(0.28, 5.31)	0.8	0.98(0.23, 4.22)	0.9
>12 y	2.97(0.23, 37.62)	0.4	0.71(0.10, 5.03)	0.7	0.18(0.04, 0.78)	0.02	0.19(0.04, 0.83)	0.03
Smoking history								
Never	1.00(Reference)		1.00(Reference)		1.00(Reference)		1.00(Reference)	
Former	0.86(0.29, 2.56)	0.8	0.50(0.16, 1.59)	0.2	0.69(0.24, 1.93)	0.5	0.38(0.13, 1.08)	0.07
Current	0.50(0.12, 2.16)	0.4	0.47(0.10, 2.08)	0.3	0.60(0.19, 1.89)	0.4	0.42(0.13, 1.34)	0.1
Alcohol intake								
Never	1.00(Reference)		1.00(Reference)		1.00(Reference)		1.00(Reference)	
Former	3.17(0.54, 18.53)	0.2	1.14(0.38, 3.86)	0.8	1.56(0.62, 3.96)	0.3	1.27(0.5.,3.22)	0.6
Current	0.61(0.21, 1.74)	0.4	0.59(0.07, 5.37)	0.6	0.64(0.13, 3.18)	0.6	0.33(0.06,1.84)	0.2
Hypertension	2.55(0.75, 8.72)	0.1	5.51(1.36, 22.27)	0.02	1.06(0.37, 3.06)	0.9	2.10(0.69, 6.41)	0.2
Diabetes	0.95(0.39, 2.31)	0.9	1.01(0.40, 2.54)	0.9	2.50(1.05, 5.97)	0.04	2.77(1.16, 6.58)	0.02
Stroke	6.59(1.73, 25.17)	0.006	5.82(1.41, 24.07)	0.02	4.19(0.83, 21.05)	0.08	6.63(1.35, 32.55)	0.02
HD vintage (per 1-mon	1.02(0.98, 1.07)	0.4	1.09(1.04, 1.14)	<0.001	1.01(1.00, 1.02)	0.005	1.01(1.00, 1.02)	0.01

increase)								
Single-pool Kt/V (per 1 increase)	0.01(0.00, 0.24)	0.003	0.01 (0.00, 0.04)	<0.001	0.42(0.06, 2.84)	0.4	0.02(0.00, 0.15)	<0.001
Hb (per 1-g/L increase)	0.99(0.95, 1.03)	0.6	0.98(0.94, 1.02)	0.3	1.01(0.98, 1.04	0.6	1.01(0.98, 1.04)	0.7
ALB (per 1-g/L increase)	0.98(0.79, 1.21)	0.8	0.88(0.71, 1.07)	0.2	1.03(0.89, 1.19)	0.7	0.96(0.83, 1.11)	0.6
CRP (per 1-mg/L increase)	1.03(0.94, 1.14)	0.5	0.96(0.86,1.07)	0.5	1.01(0.97, 1.05)	0.6	0.97(0.92, 1.02)	0.2
iPTH (per 1-pg/mL increase)	1.00(0.99, 1.00)	0.8	1.00(0.99, 1.01)	0.9	1.00(0.99, 1.01)	0.9	1.00(0.99, 1.01)	0.2

Table S2. Risk factors for cognitive impairment stratified by hemodialysis vintage. *Models were adjusted for age, sex, education level, smoking history, alcohol intake, comorbidities, hemodialysis vintage, Kt/V and the serum level of Hb, ALB, CRP, and iPTH. *Abbreviation*: OR, odds ratio; CI, confidence interval; HD, hemodialysis; Kt/V, an indicator for evaluating dialysis adequacy; Hb, hemoglobin; ALB, albumin; CRP, C-reactive protein; iPTH, intact parathyroid hormone.

Variables	single-pool Kt/V <1.2 (n=195)				single-pool Kt/V ≥1.2 (n=418)			
	Mild cognitive impairment (n=65)		Major cognitive impairment (n=104)		Mild cognitive impairment (n=163)		Major cognitive impairment (n=164)	
	OR (95% CI) *	P	OR (95% CI) *	P	OR (95% CI) *	P	OR (95% CI) *	P
Age (per 1-y increase)	7.89(1.42, 44.03)	0.02	8.30(1.49, 46.31)	0.02	1.06(1.02, 1.10)	0.005	1.08(1.04, 1.13)	<0.001
Male sex	7.26(0.34, 36.50)	0.08	3.07(0.33, 17.70)	0.09	0.95(0.53, 1.69)	0.9	1.05(0.58, 1.88)	0.9
Education level								
<6 y	1.00(Reference)		1.00(Reference)		1.00(Reference)		1.00(Reference)	
6~12 y	0.01(0.00, 5.47)	0.2	0.00(0.00, 1.25)	0.06	1.10(0.31, 3.98)	0.9	0.85(0.24, 2.99)	0.8
>12 y	2.90(0.00, 9.73)	0.8	1.17(0.00, 5.45)	0.9	0.26(0.07, 0.99)	0.04	0.30(0.08, 1.10)	0.07
Smoking history								
Never	1.00(Reference)		1.00(Reference)		1.00(Reference)		1.00(Reference)	
Former	10.97(0.09, 85.98)	0.3	7.79(0.07, 38.99)	0.4	1.18(0.52, 2.71)	0.7	0.65(0.27, 1.53)	0.3
Current	0.16(0.00, 32.05)	0.5	0.20(0.00, 41.88)	0.6	0.62(0.25, 1.53)	0.3	0.40(0.16, 1.04)	0.06
Alcohol intake								
Never	1.00(Reference)		1.00(Reference)		1.00(Reference)		1.00(Reference)	
Former	0.41(0.13, 13.25)	0.6	0.59(0.02, 19.71)	0.8	0.81(0.32, 2.07)	0.7	0.37(0.13, 1.02)	0.06
Current	0.67(0.35, 4.97)	0.5	1.56(0.55, 3.64)	0.2	0.91(0.16, 4.13)	0. 3	1.50(0.20, 11.43)	0.7
Hypertension	0.07(0.00, 9.37)	0.3	0.10(0.00, 12.98)	0.3	1.72(0.89, 3.31)	0.1	1.74(0.90, 3.36)	0.1
Diabetes	3.89(1.61, 8.46)	0.04	7.82(1.51, 11.42)	0.04	3.04(1.05, 8.79)	0.04	4.47(1.57, 12.73)	0.005
Stroke	0.41(0.01, 13.25)	0.6	0.59(0.02, 19.71)	0.8	1.01(0.48, 2.11)	0.9	0.76(0.36, 1.63)	0.5
HD vintage (per 1-mon increase)	1.11(1.01, 1.22)	0.04	1.11(1.01, 1.23)	0.03	1.01(1.01, 1.02)	<0.001	1.01(1.01, 1.02)	<0.001
Single-pool Kt/V (per 1 increase)	0.13(0.01, 4.41)	0.2	0.13(0.00, 9.50)	0.2	0.67(0.10, 4.35)	0.7	0.03(0.00, 0.24)	0.001
ALB (per 1-g/L increase)	6.71(0.00, 28.37)	0.9	5.40(0.00, 22.84)	0.9	0.94(0.83, 1.07)	0.4	0.95(0.83, 1.08)	0.4
CRP (per 1-mg/L increase)	0.01(0.00, 2.85)	0.9	0.01(0.00, 9.85)	0.9	1.01(0.97, 1.04)	0.8	0.98(0.93, 1.03)	0.4

iPTH (per 1-pg/mL increase)	1.01(0.00, 7.90)	0.9	1.01(0.02, 7.79)	0.9	1.00(0.99,1.02)	0.9	1.00(1.00, 1.03)	0.06

Table S3. Risk factors for cognitive impairment stratified by single-pool Kt/V. *Models were adjusted for age, sex, education level, smoking history, alcohol intake, comorbidities, hemodialysis vintage, Kt/V and the serum level of Hb, ALB, CRP, and iPTH. *Abbreviation*: OR, odds ratio; CI, confidence interval; HD, hemodialysis; Kt/V, an indicator for evaluating dialysis adequacy; Hb, hemoglobin; ALB, albumin; CRP, C-reactive protein; iPTH, intact parathyroid hormone
